# The neurobiology of Etruscan shrew active touch

**DOI:** 10.1098/rstb.2011.0160

**Published:** 2011-11-12

**Authors:** Michael Brecht, Robert Naumann, Farzana Anjum, Jason Wolfe, Martin Munz, Carolin Mende, Claudia Roth-Alpermann

**Affiliations:** BCCN, Humboldt University BerlinPhilippstrasse 13, House 6, 10115 Berlin, Germany

**Keywords:** whisker, vibrissa, active touch, barrel cortex, shrew, *Suncus etruscus*

## Abstract

The Etruscan shrew, *Suncus etruscus*, is not only the smallest terrestrial mammal, but also one of the fastest and most tactile hunters described to date. The shrew's skeletal muscle consists entirely of fast-twitch types and lacks slow fibres. Etruscan shrews detect, overwhelm, and kill insect prey in large numbers in darkness. The cricket prey is exquisitely mechanosensitive and fast-moving, and is as big as the shrew itself. Experiments with prey replica show that shape cues are both necessary and sufficient for evoking attacks. Shrew attacks are whisker guided by motion- and size-invariant Gestalt-like prey representations. Shrews often attack their prey prior to any signs of evasive manoeuvres. Shrews whisk at frequencies of approximately 14 Hz and can react with latencies as short as 25–30 ms to prey movement. The speed of attacks suggests that shrews identify and classify prey with a single touch. Large parts of the shrew's brain respond to vibrissal touch, which is represented in at least four cortical areas comprising collectively about a third of the cortical volume. Etruscan shrews can enter a torpid state and reduce their body temperature; we observed that cortical response latencies become two to three times longer when body temperature drops from 36°C to 24°C, suggesting that endothermy contributes to the animal's high-speed sensorimotor performance. We argue that small size, high-speed behaviour and extreme dependence on touch are not coincidental, but reflect an evolutionary strategy, in which the metabolic costs of small body size are outweighed by the advantages of being a short-range high-speed touch and kill predator.

## 1. Introduction

### (a) Purpose of the review: neurobiology of shrew active touch

**Figure 1.**
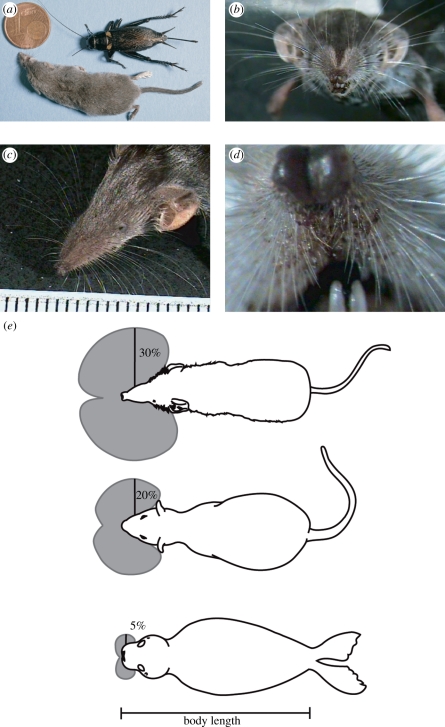
The Etruscan shrew and its vibrissal system. (*a*) An Etruscan shrew and a field cricket. The € cent coin is 16.25 mm in diameter. (*b*) Frontal view of the head of a shrew. (*c*) Etruscan shrew whisker array; the longest shrew macrovibrissae are *ca* 12 mm long. The scale shows millimetres. (*d*) High magnification view of the microvibrissae surrounding the mouth. (*e*) Schematics of vibrissal sensing volumes [1] (grey) and body size in the Etruscan shrew, the rat (middle) and the harbour seal (bottom). The percentage value refers to the length the longest facial vibrissa and states the percentage of body length that this whisker reaches.

The purpose of this review is to summarize our advances on Etruscan shrew active touch and put them in perspective with other findings on the tactile behaviour of other mammals. Specifically, we will portray the Etruscan shrew as short-range high-speed hunter. Shrews tackle a complex task: in darkness they detect, overwhelm and kill their insect prey, a fast moving target that is almost as big as the shrew itself (figure 1*a*). Crickets are abundant in shrew natural habitats [2] and are nocturnal, highly mobile animals endowed with a variety of mechanosensitive organs that mediate escape behaviours [3]. Thus, the behavioural ecology of shrews and crickets predisposes them towards interacting via sophisticated tactile behaviours.

### (b) Sensory implications of small body size

The Etruscan shrew, *Suncus etruscus*, is not only the smallest shrew, but presumably also the smallest terrestrial mammal. The adult body mass of individuals caught from their natural environment ranges from 1.6 to 2.4 g [4]. While many authors have focused on the metabolic implications of small body size in mammals, few have considered the sensory implications of body size. Here we argue, however, that such scaling relationships have important implications for vibrissal touch. Etruscan shrews have a prominent vibrissae array (figure 1*b*–*d*). In figure 1*e*, we highlight the scaling relationship of body and vibrissa length for diverse mammals, which are all thought to be ‘vibrissal’ experts. It is obvious that whiskers are relatively longer in smaller mammals; i.e. small animals have a relatively larger vibrissal sensing volume [1]. It may thus not be surprising that Etruscan shrews, like rodents, depend primarily on their well-developed array of mystacial vibrissae for tactile information [5–7]. If whiskers are protracted, the tips of the whiskers precede the snout by up to 1 cm, which is around a fifth to a third of the total body length. Etruscan shrew whiskers thus sense a longer distance ahead than in the rat or mouse. This may be necessary to avoid obstacles as the shrew locomotes very rapidly [8]. Furthermore, a longer whiskertip to mouth distance may be advantageous both in the spatial and temporal domain for successful shrew corrective manoeuvres observed in response to fast prey escape attempts [6,7]. The relatively large sensing volume scanned by the vibrissae allows the Etruscan shrew to sample more of its direct surround, which may partly explain why shrews act so fast during prey capture.

Shrews have very small eyes [9,10]. It has been suggested that owing to poor development of the eyes and visual system vision functions merely to discriminate light intensity [11–13], although in our hands, visual cues appeared to trigger defensive reactions. However, we never observed any evidence that visual stimuli (i.e. crickets behind a glass screen) trigger hunting behaviours.

Shrews have very small cochleas with only about 300 sensory hair cells in the smallest shrews investigated so far [14] and correspondingly small brain areas involved in auditory sensation [14–16]. As a result of reduced visual and auditory capacities in most shrews, olfactory and somatosensory modalities have become a highly-developed and important part of their sensory repertoire [5,17–19].

Sensory organs and the brain claim high energetic costs (for a review see Niven & Laughlin [20]). Many sensory modalities such as active whisker touch require muscular movements, which further increase the energetic costs. Hence, sensory and nervous systems are subject to two conflicting selective pressures: the need to minimize energy consumption and to generate adaptive behaviour under changing environmental conditions. More specifically, in sensory systems, there will be a trade-off between the energetic costs of a sensory structure encoding a particular modality and the amount of reliable information obtained. Trade-offs may also occur *between* sensory systems: animals with a subterranean lifestyle such as the star-nosed mole have a highly developed mechanosensory modality with sensory specializations and expanded cortical areas at the expense of reduced vision with minute eyes and a small visual cortical region [21]. Similar trade-offs have been documented in the naked mole rat, which has been shown to be completely blind and possesses specialized sensory hairs along the body that might guide its movement within tunnels [22,23].

### (c) Metabolic selection pressure for efficient sensorimotor performance

Small body size does not only directly impact on vibrotactile sensing, but it also results in unique selection pressures on the Etruscan shrew's hunting behaviour. Because of their small size and their large surface to volume ratio, Etruscan shrews have an extraordinarily high-energy turnover. This presents an extreme challenge to all functions of the body, including respiration, oxygen transport, muscle parameters, but most importantly here the sensory and neural systems. Only because Etruscan shrews are highly efficient hunters, are they able to meet these extreme metabolic demands.

Etruscan shrews are usually homoeothermic with a normal body temperature between 34°C and 38°C [4,24]. Etruscan shrews resting at an ambient temperature of 20°C have a mean body temperature of 34.7±0.5°C [24]. During activity, the mean body temperature is 2°C–3°C higher than at rest [24,25]. However, in case of food restriction and at low ambient temperature, they can reduce their body temperature and enter a torpid state to cut down their resting energy expenditure. Torpor is defined as a state of decreased physiological activity, usually characterized by a reduced body temperature and reduced metabolism. In laboratory conditions, daily torpor cycles were observed with body temperatures lowered to about 12°C, in extreme cases even to 6°C [26,27]. Shrews can warm up from torpor very rapidly at a rate of around 1°C per minute by muscle shivering and heat generation from brown adipose tissue [27]. Under normothermic resting conditions, the specific oxygen consumption rate of *S. etruscus* is 67 times higher than in humans. A maximal heart rate of up to 1500 beats per minute exceeds all values reported for other endothermic animals [4]. This species has the highest mass-specific metabolic rate of all mammals [28] and thus there is an immense pressure to obtain prey. Shrews as small as *S. etruscus* need to ingest food at least every hour and have to consume up to six times their own body weight of insects every day [29].

Muscles play a major role in the capture and chewing of prey, but *S. etruscus* requires fast skeletal muscles not only for locomotion but also for effective heat production and for an extremely high ventilation rate [28]. Skeletal muscles can contract at up to 780 min^−1^ for running, up to 3500 min^−1^ for shivering and up to 900 min^−1^ for respiration. Both structural and functional properties demonstrate that the Etruscan shrew's skeletal muscles are well adapted to fit the needs of this animal's extreme metabolism; they lack slow-twitch type I fibres and consist only of fast-twitch IID fibres. The enzymatic characteristics of these fibres make them optimally equipped for an almost purely oxidative metabolism [30].

With a brain mass of about 60 mg, the Etruscan shrew has the smallest mammalian brain known [31]. In such small brains, axons are typically densely packed, small in diameter and mostly unmyelinated. Unmyelinated axons have high capacitance per unit length and are energetically more expensive than myelinated axons [32]. In the tiny shrew, the estimated metabolic cost for generating an action potential for all white matter fibres averaged is an order of magnitude higher than in the macaque and 97 per cent of this cost is accounted for by the unmyelinated axons.

In summary, surface to volume considerations imply that a homoeothermic body temperature is metabolically highly costly. The musculature of Etruscan shrews is specialized for fast movement, and an increased body temperature might also offer massive advantages in terms of processing speed (see below).

### (d) Sensory ecology: Etruscan shrews specialize in a hidden life in slits

There are more than 300 species of shrews and they share common features, such as a small body size and prominent whiskers on a pointed snout. Shrews belong to the order Soricomorpha. Fossil evidence suggests that the earliest mammals were shrew-like in body size. Their brains were similarly small as those of extant shrews and possibly afforded similar behavioural capabilities [33,34].

In Europe, the Etruscan shrew is mainly found in the Mediterranean lowlands and in Asia in a belt extending between 10° N and 30° N [30]. Their habitat includes forest, shrub and grassland environments [35]. As for many other species in the genus *Suncus*, the Etruscan shrew is most likely solitary and territorial, except during the breeding season [36,37]. Being hunted by predator birds such as owls [38], shrews try to avoid moving uncovered in the open field, but rather seek shelter under piles of rock, pieces of bark or other organic material and in tunnels that they dig in loose soil. Often they are found resting in old dry stone walls, where they also build nests for bringing up their young. Strikingly agile animals, shrews squeeze their body through tiny holes and they are able to enter and capture prey in slits as thin as 7 mm.

Shrews are opportunist insectivores and all species consume a wide range of prey. Studies of the feeding habits of shrews and their prey availability demonstrate that small size brings benefits as well as costs [39]. The greatest advantages for small shrews are their lower absolute food requirements and the ability to subsist on small, numerous and accessible arthropods with high encounter rates, available in different seasons and low-productivity habitats. Major costs of small size are a reduction in food niche breadth and prey biomass resulting from restrictions on the type and size of prey eaten, and large territory requirements with a consequential increase in the energetic cost of foraging and territory maintenance. Owing to their constant food requirement, shrews have polyphasic circadian activity patterns with frequent activity bouts distributed evenly over a period of 24 h [9,35,40–43]. This means that shrews have to be able to successfully hunt in twilight as well as in darkness. Vision can be furthermore limited in typical shrew habitats, such as dense brush vegetation or tunnels in stone walls or the soil [9] and indeed, sight only seems to play a minor role for navigation and prey capture [6,7,13,44].

When exploring new environments, shrews frequently emit faint, high-pitched laryngeal calls (‘twittering’) of unclear function [9]. While a few authors claimed that shrews make use of echolocation [45–48], others found no evidence for this ability [8,19]. A recent study proposed that shrew-like calls can yield echo scenes useful for habitat assessment at close range, beyond the range of the shrews' vibrissae. At the same time, it seems unlikely that they can make bat-like use of echolocation to search for prey [49]. In summary, we suggest that the secret life of Etruscan shrews in slits, where they hunt large and diverse prey, might predispose them to rely on proximal tactile cues.

## 2. Tactile prey capture behaviour

### (a) Tactile hunting of highly mechanosensitive cricket prey

Since the pioneering work of von Uexküll, it is clear that understanding sensory performance of predators requires analysis of the sensory characteristics of their prey [50]. Crickets are found in abundance in the natural habitat of the Etruscan shrew and therefore are thought to be an important prey [2]. A cricket can measure up to 35 mm (body of the Etruscan shrew measures between 35 and 50 mm) and has very long antennae and prominent jumping legs (figure 1*a*). Crickets are fast moving prey and very capable of evading attacks. A variety of mechanoreceptors, different kinds of receptors and mechano sensory sensilla are found in and on the cricket body and appendages, just as in other insects (e.g. cockroaches, locusts). A cricket shows a range of behavioural responses to stimulation of its mechanoreceptors, extending from ignoring the stimulus to altering complex behavioural sequences such as avoidance manoeuvres, orientation and approach or fighting (see [51,52]). Furthermore, the input from mechanoreceptors is known to inhibit ongoing behavioural activity, e.g. singing or walking stops when a predator approaches [53]. While the antennae are versatile head appendages with the ability to sense the environment up to twice its body length, cerci, the two caudal antenna-like appendages are mainly known to guard the rear of the insect [54–57]. Any defensive or escape behaviour guided by cercal mechanoreceptors depends on their stimulation [58].

Mechanisms of escape behaviour have been studied intensely [3,59,60]. Wind and touch stimuli have been used to study defensive (kicking) and escape responses. When a digger wasp makes contact with the cricket, it first leads to a head stand (sudden raising of the abdomen), followed by a stilt stand with the further raising and tilted posture, which is followed by a rapid kick with one hind leg casting the wasp several centimetres away. The kick is completed in 100 ms after the touch and can also be followed by a second kick [52,61]. We observed that Etruscan shrews quickly retracted their snout after placing attacks on crickets, probably to avoid being kicked. The escape response can be a turn, a jump or both and often is followed by running.

### (b) Tactile guidance of prey capture

**Figure 2.**
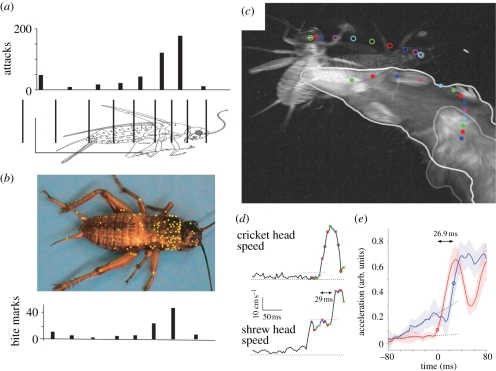
Precision and speed of shrew attacks. (*a*,*b*) Shrew attacks are selectively placed on the thorax of crickets. Modified from Anjum *et al*. [6]. (*a*) Attack histogram derived by analysing video sequences (*n* = 450 shrew attacks on approx. 130 crickets). (*b*) Bite mark positions (yellow squares superimposed on a cricket photograph (*n* = 94 bite marks on 25 freshly killed, immobilized or injured crickets)) and bite mark histogram. (*c*–*e*) Mid-attack change of direction. Modified from Munz *et al*. [7]. (*c*) Still frames from before and at the end of the attack (time lapse between images = 0.23 s) are overlaid. Dots and circles are the head positions of the shrew and cricket, respectively. Dots and circles are colour-coded for simultaneous head positions of the shrew and cricket. (*d*) Head speed of the cricket (top) and shrew (bottom). Note the 29 ms lapse between the cricket's speed increase and the shrew's speed increase. Black dotted lines represent 0 cm s^−1^, red-dotted lines represent the thresholds used to determine the time of speed increase. (*e*) Average of six such attacks. Black dashed lines are linear fits to the baseline acceleration prior to the sudden increase in cricket acceleration (*t* = 0 ms). Shaded regions represent ±1 s.e. The difference in time between the cricket and the shrew acceleration increase was 27 ms. (*e*) Brown curve, cricket; blue curve, shrew.

Anjum *et al*. [6] studied the hunting behaviour of Etruscan shrews in a laboratory setting. In these experiments, the spatio-temporal analysis of numerous attacks was combined with whisker removal and prey manipulation experiments. Etruscan shrews direct their attacks selectively to the cricket's thorax and manage to keep this precision regardless of the size of the prey (figure 2*a,b*). They attack crickets from the side with a narrow distribution of attack angles around 90° relative to the cricket's body axis. Although most attacks are directed straight ahead, there is a slight lateralization in the hunting behaviour towards rightward attacks.

#### (i) High speed of prey capture

Prey capture occurs very quickly, i.e. in 80–200 ms per attack, with short inter-attack intervals. While first attacks were distributed relatively broadly over the cricket's body, subsequent attacks were directed more and more precisely to the thorax.

#### (ii) Whisker-dependent shape recognition

Removal experiments showed that both macro- and microvibrissae are required for hunting [6]. Experiments with dummy prey objects showed that shrews attacked a plastic replica of a cricket but not other plastic objects of similar size. Altering the shape of crickets by gluing on additional body parts from donor animals revealed that the jumping legs but not the head are key features in prey recognition. Addition of such ‘ectopic’ jumping legs is highly confusing for shrews and leads to dramatic changes in attack patterns. Thus, tactile shape cues are both necessary and sufficient for evoking attacks. Both the generalized effects of cricket shape manipulation experiments and characteristics of corrective manoeuvres indicate that shrew behaviour is guided by Gestalt-like prey descriptions [6].

### (c) Shrew whisking and active touch in prey capture

We recently characterized Etruscan shrew whisking and tactile behaviour during prey capture [7]. To this end, we combined staged shrew–cricket encounters with whisker tagging and high-speed videography.

#### (i) Basic characteristics of Etruscan shrew whisking

Like other mammals, such as mice and rats, Etruscan shrews engage in rhythmic back and forth whisker movements, i.e. whisking. The average power spectrum shows a very clear peak in the shrew whisking at approximately 14 Hz. This is a considerably higher whisking frequency than that of rats (approx. 8 Hz), but is similar to mice [62]. Clearly, shrews employ periodic whisking during their hunting behaviour and the shrew whiskers are under active muscle control. Compared with rats, shrews had lower amplitude whisking (approx. 30° versus approx. 50° in rats). Interestingly, as in rats, retraction velocity was almost double protraction velocity.

#### (ii) Whisking during hunting

Etruscan shrew whisking during hunting can be divided into phases: (i) immobile resting prior to hunting. Prior to hunting shrews often showed very little head or whisker movement. (ii) Search phase. The beginning of the search phase was determined by an increase in the head velocity. Concurrent with increased head motion the whisker set angle increased and whisker motion increased. During the search phase we often observed highly regular periodic whisking. (iii) Contact phase. The first whisker-to-cricket contact defined the transition from the search to the contact phase. This phase was kept very short by the shrew, as crickets tried to escape before the shrew was able to strike. Following contact, whisking amplitude decreased and there was a small increase in the whisker set angle. (iv) Attack phase. Attack was defined by a sudden increase in head acceleration directed towards the cricket. This is a brief behavioural event with a sharp increase in head acceleration. The shrew's trunk dramatically bent during the strike and assumed the shape of a parrot beak.

#### (iii) Mid-flight changes in attack direction indicate short reaction times

As illustrated in figure 2*c*–*e*, we found that shrews were able to react to cricket movements during the short duration of the attack. In figure 2*c*, we overlaid video images taken just before and at the end of an attack. The dots and circles show the head positions of the cricket and shrew, respectively, during the attack. The dots and circles are colour coded to show simultaneous shrew and cricket head positions (note that the first four cricket head positions are nearly identical). In this example, the shrew is initially moving upward in the video and the cricket is still. When the cricket suddenly jumps backward, the shrew reacts by adjusting its trajectory. We estimated the reaction time of the shrew by looking at the time delay between the cricket's sudden speed increase, corresponding to its attempted flight, and the shrew's increase in head speed as it adjusts its attack. In this example, we found that it took the shrew only 29 ms to react to the cricket's escape attempt (figure 2*d*). On average, the shrew's increased head acceleration followed the cricket's sudden acceleration by 27 ms (figure 2*e*). It was previously reported that shrews react to underwater stimuli with a latency on the order of 20 ms, in good agreement with the values reported here [19]. Overall, the observations from high-speed videography strongly support the idea that shrews out-manoeuvre their very large prey by high-speed performance. Indeed, about 40 per cent of shrew attacks target stationary prey and the first strike often occurs prior to any evasive manoeuvre [6]. Both short reaction times and short attack intervals suggest that shrews identify and target their prey with a single touch.

## 3. The shrew somatosensory system

### (a) Periphery

**Figure 3.**
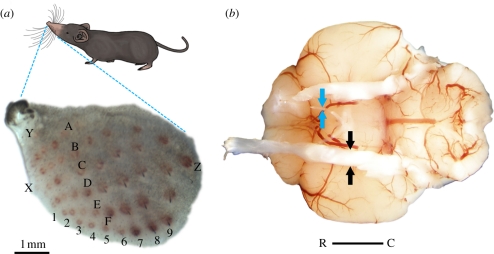
Periphery of the Etruscan shrew vibrissal system. (*a*) Pattern of vibrissal follicles shown in a flattened preparation showing six rows (A–F). Each row contains six to nine whiskers. In addition, there are three whiskers not contained in a row or an arc and labelled X, Y and Z. Scale bar applies to (*a*) and (*b*). (*b*) Ventral view of the shrew's brain. The trigeminal nerve is indicated as black arrows and optic nerve as blue arrows. R, rostral; C, caudal.

The Etruscan shrew's prominent whisker fan has already been introduced in figure 1. Selective whisker removal experiments demonstrated a functional differentiation of shrew whiskers in prey capture [6]. The large macrovibrissae (figure 1*c*) were required for prey targeting, whereas the small microvibrissae around the shrew's mouth (figure 1*d*) were necessary for initiating the final strike/bite in attacks. Figure 3*a* shows the left whiskerpad with the vibrissal follicles clearly visible. The whiskers on each side of an Etruscan shrew's snout are arranged in a grid made up of six rows (A to F) and several arcs. Each row contains six to nine whiskers. In addition, there are three whiskers not contained in a row or an arc, labelled X, Y and Z. In total, there are more than hundred vibrissae extending like a fan from the snout of the Etruscan shrew. In larger shrews, even higher numbers of vibrissae have been described [60,63,64]. Differences between shrew and rodent vibrissal follicles have been described [63,65,66].

Touch signals from the follicles are relayed to the brainstem via the trigeminal nerve. In the long-clawed shrew (*Sorex unguiculatus*), which is about 10 times larger than the Etruscan shrew, each sinus hair follicle is innervated by 60–100 myelinated fibres [61]. The fibres from the sinus hair follicles join to form the maxillary branch of the trigeminal nerve. The thick trigeminal nerve compared with the much thinner other cranial nerves gives a stunning impression of the significance of touch information from the facial region for the Etruscan shrew. Figure 3*b* shows the trigeminal nerve framed by black arrows and the optic nerve framed by blue arrows for comparison. The differences in macromorphology of the sensory cranial nerves are mirrored by the number of sensory fibres contained in those nerves. In the northern short-tailed shrew (*Blarina brevicauda*), there are about 15 000 fibres in the infraorbital part of the trigeminal nerve compared with less than 500 fibres in the optic nerve [67]. More recently, differences in fibre distribution were reported for the similarly sized American water shrew (*Sorex palustris*), which has about 30 000 axons in the trigeminal nerves, less than 6000 in the optic nerves and 6000–7000 in the auditory nerves [68]. The most extreme of the small mammals is probably the star-nosed mole (*Condylura cristata*)—its touch sensitive appendages are innervated by about 100 000 myelinated nerve fibres [69].

### (b) Cortical organization: anatomy

The Etruscan shrew has the smallest brain of all mammals. Its cerebral cortex is very thin, only 400 to 500 µm on average [16,70]. As in other mammals, the cortex of the Etruscan shrew is a cytoarchitectonically heterogeneous sheet of tissue. The presence of distinct cortical areas is suggested by the fact that different staining methods (Nissl, cytochrome oxidase activity, myelin) indicate the same areal borders. Sensory neocortical areas could be clearly identified by cytochrome oxidase and myelin staining in coronal and tangential brain sections. In total, there are about 10–15 cortical areas—a relatively large number given the small size of the Etruscan shrew cerebral cortex [71,72].

We compared volumes of cortical areas of the Etruscan shrew with data for the cerebral cortex of the rat, which is 100 times larger than in the shrew. We included all areas of the neocortex, as well as entorhinal and piriform cortex [72,73]. The most striking difference is that entorhinal cortex and piriform cortex comprise a much larger part of the cortical mantle in the Etruscan shrew (approx. 42%) than in the rat (approx. 17%). We recorded neuronal responses to touch stimuli in somatosensory cortex, insular cortex and perirhinal cortex of the Etruscan shrew (see §3*c*), these areas combined take up about one-third of the total cortical volume in the Etruscan shrew as well as in the rat. In the Etruscan shrew auditory and visual cortex comprise only about 2–3% of the cortical volume, whereas it is four to five times more in the rat. The differences in relative cortical volumes are mirrored by findings in relative cortical area sizes [74] and neuron numbers [72]. In summary, the Etruscan shrew devotes a large cortical volume to somatosensation, whereas visual and auditory processing takes up only small fractions. The superb tactile capacities are reflected in the anatomy of the shrew cortex.

### (c) Cortical organization: physiology

The neurophysiology of the Etruscan shrew is of interest both because of their small brain size and their remarkable behavioural capacities. Work on related northern American shrew species showed that these shrews have few sensory cortical areas, which include a large primary and secondary somatosensory cortical area and a primary visual and auditory cortex [15], a pattern in line with the numerous specializations of insectivores for somatosensation [21].

**Figure 4.**
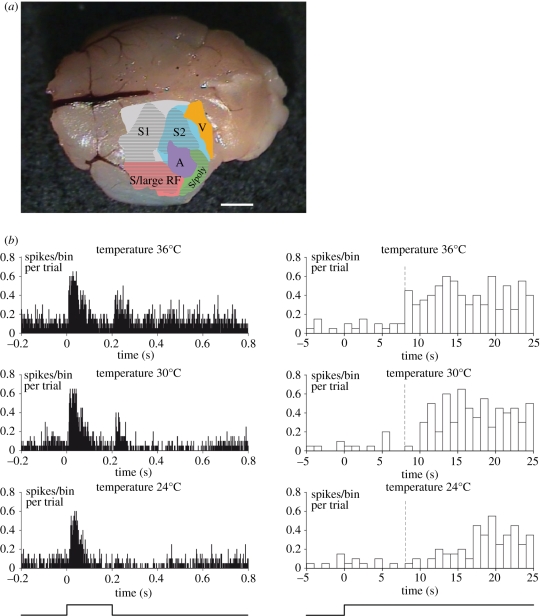
Etruscan shrew neurophysiology. (*a*) Physiologically derived map of the Etruscan shrew cortex. An average map of cortical regions was delineated by electrophysiological mapping experiments. Dotted areas indicate macrovibrissae responses. S1, primary somatosensory cortex; S2, secondary somatosensory cortex; V, visual cortex; A, auditory cortex; S, somatosensory; RF, receptive field. (*b*) Whisker responses at different shrew body temperatures. Peristimulus time histograms (1 ms bin size) of multi-unit responses to piezoelectric stimulation (approx. 10° deflection and approx. 1 ms rise time) of a single whisker (*n* = 20 trials per temperature). Neuronal responses were obtained at the same recording site in putative area S1 while the body temperature of the shrew varied from 36°C (top) to 24°C (bottom). Stimulus onset at time point 0, duration of the stimulus 200 ms, as indicated below. Right: same data as left, but zoomed into the time of stimulus onset. Dashed line indicates the time of first response at 36°C. Note the latency changes with temperatures.

We investigated cortical organization in Etruscan shrews by electrophysiological mapping in combination with histological verification of recording sites [16]. We characterized cortical multi-unit responses to auditory, visual and somatosensory stimuli. We found that large parts of shrew cortex (7.3 mm^2^ of approx. 12 mm^2^ total neocortical surface, i.e. approx. 60%) responded to such stimuli (figure 4*a*). The true fraction of sensory cortex in Etruscan shrews is probably substantially higher, because we did not test for olfactory and gustatory responses and we could only map three-quarters of the cortical sheet.

Auditory and visual stimuli activated only small parts of Etruscan shrew cortex (figure 4*a*). Tactile processing, however, appears to occur in multiple cortical regions. Large fractions of these somatosensory areas responded to macrovibrissae stimulation. We identified two topographically organized somatosensory areas with small receptive fields referred to as putative primary somatosensory cortex (S1) and putative secondary somatosensory cortex (S2). A third tactile region was located posterior-laterally, where we observed large somatosensory receptive fields and often polysensory responses. Furthermore, we identified an anterior-lateral region with large unimodal somatosensory receptive fields. The latter two regions partially overlapped with piriform cortex. Putative S1 and S2 have relatively small receptive fields. The receptive field size of about 10 whiskers per multi-unit recording site was similar or slightly larger than what has been measured in other shrew species [15] and larger than most of the receptive field sizes reported in rodent S1 [75]. It appears probable that putative areas S1 and S2 of the Etruscan shrew are homologous to these respective areas described in other shrews and to areas S1 and S2 in rodents. If one compares the Etruscan shrew cortex to that of other mammals studied thus far, it is clear that this animal is one of the most extreme tactile specialists studied to date. Only a few animals such as the star-nosed mole [69] and the naked mole-rat [76] devote a similar fraction of their neocortex to somatosensory representations.

We also investigated the effects of body temperature on cortical processing. These experiments were carried out under urethane anaesthesia. In order to minimize the stress associated with the intraperitoneal urethane injection, animals were first lightly anaesthetized by isoflurane inhalation. We then injected millilitres of 4 per cent 145 urethane solution in water for an adult approximately 2.5 g shrew (a dose of approx. 0.7 mg urethane g^−1^ body weight). To this end, we made use of the fact that shrews easily withstand passive cooling during anaesthesia. Their resistance to cooling might be related to their physiological ability to reduce body temperature and enter a torpid state (see §1*c*; [26,27]). At a low body temperature of 24°C neuronal response, latencies to whisker stimulation with a piecoeletric device were long and responses to stimulus offset were absent (figure 4*b*). With increasing body temperatures, latencies became faster and the off-response was more prominent (figure 4*b*). Thus, even in a torpid state with low body temperature, the cortex responds reliably to sensory stimulation, albeit with longer latencies. The exact reason for the massive increase in response latency is not yet clear. Both neural (decreased axonal transduction velocity and delays in synaptic transmission) and biomechanical (changes in tissue/whisker biomechanics through altered viscous damping or slack) factors could play a role. The available data suggest that transduction of force is mediated directly by force gated channels in rodents and occurs at very short latencies (down to 300 µs) [77,78]. Furthermore, the speed of mechanotransduction appears to be relatively insensitive to temperature [79]. These considerations point to a neural origin of the increase in response latency.

With their specialized musculature and their extraordinarily high content in brown adipose tissue, Etruscan shrews possess effective mechanisms for thermogenesis, which allow them to heat up very quickly. During activity, body temperature can climb to 38°C. For a non-mammalian insect-hunting species, the diurnal basking lizard *Lacerta vivipara*, it was shown that maintaining high body temperatures of 30°C–36°C increased predatory efficiency [80]. Thus, homeothermy might offer the shrew considerable temporal advantage over its poikilothermic prey, which operates at lower temperatures.

## 4. Discussion

Comparing shrew touch to tactile sensing in other mammals, we find both similarities and striking differences. Human haptic sensing (reviewed in this volume by Klatzky & Lederman [81]) is similar in that it also extracts shape information from touch. A striking difference is the speed of performance, which is slow (compared with vision) in the human haptic system, because it relies on the serial/gradual scanning of objects with fingertips [81]. This is very different from vibrissal touch, in which global stimulus features appear to be extracted in a single whisker sweep. Shrew whisker touch is breathtakingly fast such that prey capture movies need to be slowed down several-fold to be accessible for visual analysis. Two factors might contribute to this difference in speed. First, sensing volume [1] is—compared to body size—large for shrew whiskers, but relatively small for fingertips. Second ecological constraints on tactile sensing are very different in shrews and humans. Shrews use whiskers to hunt fast moving prey, which is not the case for human finger use. The Etruscan shrew's tactile prey recognition shares characteristics of human visual object recognition [6]: (i) it is size invariant, (ii) motion-invariant, (iii) it is based on Gestalt-like prey descriptions. We refer to prey sensing in shrews as Gestalt-like, because they do seem to analyse prey not in a piecewise fashion, but instead seem to form a global construct of what a cricket is like. As a consequence, ‘local’ manipulations of prey shape such as the addition of another pair of jumping legs can have ‘global’ effects and result in changes of most shrew attacks on such manipulated prey. It is not yet known why shrews rely on such Gestalt-like prey descriptions. We argue that Gestalt-like prey descriptions help them in directing prey capture manoeuvres, where local information, such as a contact with the abdomen can be used to steer an attack towards the thorax. Furthermore, Gestalt-like prey descriptions might help in generalizing across prey shapes and sizes, an obvious advantage given the diverse prey that shrews hunt in their habitats.

Touch in another highly tactile insectivore—the star-nosed mole—is reviewed by Catania [82]. Similar to shrews, star-nosed moles appear to be specialized to handle prey very quickly. Different from Etruscan shrews, however, the star-nosed mole touch seems to be specialized for handling a large number of small (simple) prey items rather than to focus on sophisticated attack manoeuvres and the capturing and sensing of large prey [76].

Overall, we find that the Etruscan shrew is not only one of the smallest mammals, but also one of the fastest and most tactile mammalian hunters. These three features (small size, high-speed and extreme dependence on touch) of prey capture are hardly coincidental. Instead, we argue that the shrew has responded to the strong selection pressures associated with the metabolic costs of being a small mammal by taking full advantage of the added speed that endothermy permits. It also takes advantage of the fact that the relative vibrissal sensing volumes seem to be inversely related to body size and the very fast transduction via mechano-gated channels [77,78] in the somatosensory system.
